# Dysferlinopathy misdiagnosed with juvenile polymyositis in the pre-symptomatic stage of hyperCKemia: a case report and literature review

**DOI:** 10.1186/s12920-022-01284-y

**Published:** 2022-06-20

**Authors:** Cecilia Contreras-Cubas, Francisco Barajas-Olmos, Maria Inés Frayre-Martínez, Georgina Siordia-Reyes, Claudia C. Guízar-Sánchez, Humberto García-Ortiz, Lorena Orozco, Vicente Baca

**Affiliations:** 1 Immunogenomics and Metabolic Diseases Laboratory, National Institute of Genomic Medicine, SS, Mexico City, Mexico; 2 Department of Neurophysiology, Hospital de Pediatría, CMN Siglo XXI IMSS, Mexico City, Mexico; 3 Department of Pathology, Hospital de Pediatría, CMN Siglo XXI IMSS, Mexico City, Mexico; 4 Department of Physical Medicine and Rehabilitation, Hospital de Pediatría, CMN Siglo XXI IMSS, Mexico City, Mexico; 5 Department of Rheumatology, Hospital de Pediatría, CMN Siglo XXI IMSS, Mexico City, Mexico

**Keywords:** Dysferlinopathy, Polymyositis, Limb-girdle muscular dystrophy, Case report, Whole exome sequencing

## Abstract

**Background:**

Dysferlinopathy encompasses a group of rare muscular dystrophies caused by recessive mutations in the *DYSF* gene. The phenotype ranges from asymptomatic elevated serum creatine kinase (hyperCKemia) to selective and progressive involvement of the proximal and/or distal muscles of the limbs. Bohan and Peter criteria are the most widely used for the diagnosis of polymyositis, but they have limitations and can misclassify muscular dystrophies with inflammation as polymyositis. Most dysferlinopathy patients have muscle biopsies with inflammation and thus are vulnerable to misdiagnosis with polymyositis and inappropriate treatment with steroids and immunosuppressors.

**Case presentation:**

We describe a 14 years-old male patient who was referred for assessment of asymptomatic hyperCKemia (26,372 IU/L). An X-linked dystrophinopathy initially was ruled out by direct genetic testing. Juvenile polymyositis was considered based on muscle biopsy, creatine kinase levels, and electromyography changes. Corticosteroid treatment triggered proximal lower limb muscular weakness, and no full muscular strength recovery was observed after corticosteroid withdrawal. Based on these observations, a limb-girdle muscular dystrophy (LGMD) was suspected, and LGMDR2 was confirmed by whole exome sequencing.

**Conclusion:**

We report a dysferlinopathy patient who was misdiagnosed with juvenile polymyositis and explore in a literature review how common such misdiagnoses are. With diagnosis based only on routine clinicopathological examinations, distinguishing an inflammatory myopathy from dysferlinopathy is quite difficult. We suggest that before establishing a diagnosis of “definite” or “probable” juvenile polymyositis, according to Bohan and Peter or current ACR/EULAR criteria, a muscular dystrophy must first be ruled out.

## Background

Dysferlinopathy encompasses a group of rare muscular dystrophies caused by recessive mutations in the *DYSF* gene. This gene encodes dysferlin, a transmembrane protein found in the sarcolemma, with an essential role in plasma membrane repair [1]. Mutations in *DYSF* are associated with a wide spectrum of phenotypes, ranging from asymptomatic elevated creatine kinase (CK) in the blood (hyperCKemia) to the selective and progressive involvement of the proximal and/or distal muscles of the limbs. The two major phenotypes are limb-girdle muscular dystrophy type 2B (LGMD2B), now called LGMDR2 according to the new nomenclature [2], presenting with proximal weakness in the lower limbs, and Miyoshi muscular dystrophy-1 (MMD1), a distal myopathy initially affecting the posterior compartment muscles of the leg. Other less frequent phenotypes include the more rapidly progressive distal myopathy with anterior tibial involvement, proximodistal weakness, and pseudometabolic presentation [3]. Although rare cases of congenital and late-onset presentation have been described, muscle weakness usually occurs in the teenage years or early adulthood (on average 15–27 years). The detection of dysferlin deficiency in muscle or blood and the identification of *DYSF* mutations are the main tools for diagnosing dysferlinopathy [4]. However, some clinical characteristics of dysferlinopathies such as proximal muscle weakness, elevated serum CK, and the prominent inflammatory findings on muscle biopsy may resemble idiopathic inflammatory myopathies (IIM).

Here we present a case of misdiagnosed dysferlinopathy with juvenile polymyositis (PM) and, based on a review of the literature, including the current evidences and classification criteria for IIM and the approach to the patient with hyperCKemia, we emphasize that PM is a rare disease and that before establishing a diagnosis of "definite" or "probable" juvenile PM, according to Bohan and Peter or ACR/EULAR criteria, muscular dystrophy with inflammatory features on muscle biopsy should be considered.

## Case presentation

Here we present the case of a 14 years-old male patient in Mexico who was referred to our rheumatology department for assessment of an incidental finding of asymptomatic hyperCKemia (26,372 IU/L). There was no history of familial neuromuscular disorder or parental consanguinity or of exposure to myotoxic medications or substances. The patient engaged in 3 h daily of high-performance sports and had not experienced myalgia, cramps, or pigmenturia during or after physical activity. Initially, a neurologist considered that the hyperCKemia was related to exercise, but the patient’s CK serum levels persisted above 20,000 IU/L despite cessation of sports. Neuromuscular and systemic examination was normal, including an electromyography (EMG) and nerve conduction studies. No cardiac or respiratory complications were found. Myositis-specific (Mi2, TIF1g, MDA5, NXP2, SAE1, Jo1, SRP, PL7, PL12, EJ, OJ), myositis-associated (Ku, PM-Scl 75/100, Ro52), and antinuclear antibodies were negative. Thyroid-stimulating hormone level was normal.

**Fig. 1:**
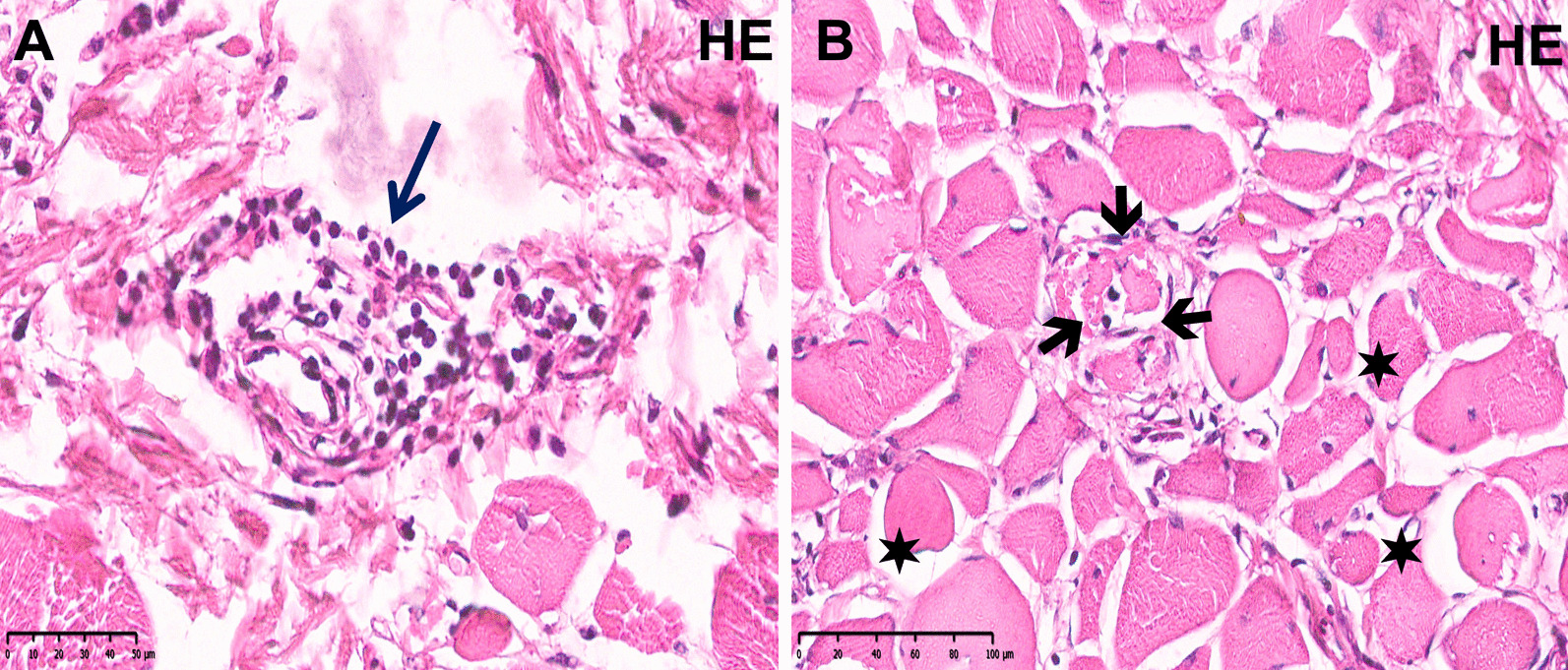
Right quadriceps muscle biopsy. Representative microscopic images of hematoxylin and eosin (HE) staining. **A** HE 40x, perivascular lymphocytic infiltrates are observed (arrow). **B** HE 30x, variation in fiber size (stars), degeneration, and necrosis is noted (arrows). These images were obtained using the following equipment: microscope BX53 and camera DP73 (Olympus, Tokyo, Japan). Scanner Hamamatsu, Nanozoomer S210-NDP. View 2 version 2.9.29, was used as acquisition software and the measurement resolution was 1200dpi

Based on the patient’s sex and serum CK levels, an X-linked dystrophinopathy was suspected, but multiplex polymerase chain reaction analysis and multiplex ligation-dependent probe amplification analysis detected no *DMD* deletions. Six months later, the patient underwent muscle biopsy from the right quadriceps that showed necrosis along with endomysial and perivascular lymphocytic infiltrates, and no fibrosis or fatty infiltration was seen (Fig. 1). A new EMG and nerve conduction studies revealed a myopathic pattern. These findings were felt to be consistent with PM.

**Fig. 2:**
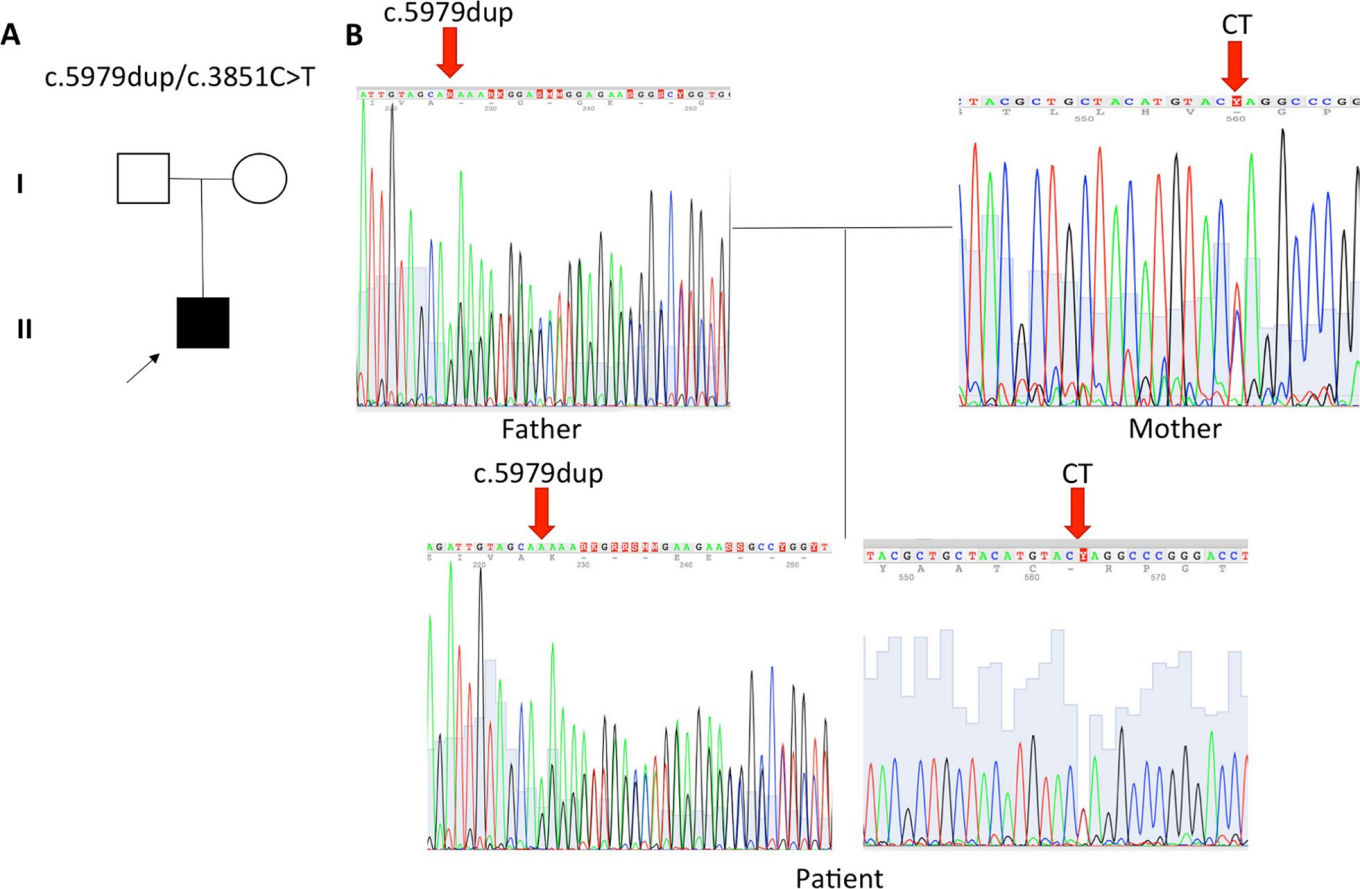
**A** Family pedigree showing the c.3851C > T and c.5979dup mutation carriers. The patient and his mother carried the c.3851C > T variant, while the patient and his father the c.5979dup variant. **B** Nucleotide chromatograms of the affected region. Red arrows indicate the variants

He was treated with intravenous methylprednisolone (IVMP) 1 g/day for 3 days and continued with prednisone 0.5 mg/kg/day and methotrexate 15 mg/weekly. Despite a decrease in CK serum levels from 28,457 to 21,671 IU/L, the patient began to experience proximal muscle weakness of both lower limbs, which worsened after a second monthly IVMP (500 mg/day for two days), with sparing of the upper limbs. Based on CK serum levels, the onset of proximal lower limb weakness after corticosteroid treatment, and the prominent inflammatory changes seen on muscle biopsy, a LGMD was suspected, and methotrexate and glucocorticoid treatment was suspended. After whole-exome sequencing (WES) (NGS; Illumina HiSeq 2500 sequencer), the diagnosis of LGMDR2 was confirmed based on a compound heterozygous variant of the *DYSF* gene. The first mutation was c.3851C > T, which causes a Gln →Ter amino acid change at position 1160 (p.Q1160X), leading to a stop codon in exon 32. The other mutation was a c.5979dup in exon 53, which causes a p.Glu1994ArgX3 frame shift. Sanger sequencing confirmed that the mother was a c.3851C > T carrier and that the father had the c.5979dup mutation (Fig. 2).

## Discussion and conclusions

The juvenile forms of IIM (age at onset < 18 years) include juvenile PM, juvenile dermatomyositis, overlap myositis, and immune-mediated necrotizing myositis [5]. There is currently no widely accepted consensus regarding the classification of IIMs. The Bohan and Peter criteria are the most widely used for the definition of PM and dermatomyositis [6, 7]. According to these criteria, a diagnosis of definite PM requires all of the following: (1) proximal muscle weakness, (2) elevated serum CK, (3) EMG changes, and (4) muscle biopsy showing inflammation. All but the muscle biopsy findings are required for the diagnosis of probable PM. However, the Bohan and Peter criteria have some limitations because they do not clearly specify how to exclude other forms of myopathy, leading to the potential for misclassification. In a recent study of 255 patients classified as having definite or probable IIM by the current EULAR/ACR criteria [8], 124 were classified as PM, but only 37 (14.5%) were classified as PM according to expert opinion [9]. Furthermore, a detailed review of these 37 cases led to only 9 (24.3%) patients remaining classified as PM, corresponding only to 3.5% (9/255) of the original cohort [10].

Over time, PM has been questioned as a distinct entity, and many of these patients may be better described as having an alternative diagnosis [11–15]. Dermatomyositis is well recognized in children, but the existence of juvenile PM has been highly debated [16, 17]. In fact, it took almost 10 years to recruit enough patients to establish the current EULAR/ACR criteria for adult and juvenile IIM, and even then, the number of children with PM was insufficient for adequate study. For this reason, pediatric rheumatology experts on the International Myositis Classification Criteria Project have recommended extrapolating the adult subclassification of IIM criteria for juvenile PM [8].

Inherited myopathies such as calpainopathy, dysferlinopathy, facioscapulohumeral muscular dystrophy, and dystrophinopathy may be associated with inflammation on muscle biopsy. They also frequently present with proximal muscle weakness, elevated CK, and EMG with a myopathic pattern. Therefore, the differential diagnosis of PM from muscular dystrophies, based upon histologic and clinical findings, may be challenging [18, 19]. In a retrospective clinicopathological analysis from Australia, for example, of 13 cases with an initial diagnosis of juvenile PM, 12 (92.3%) were found to be muscular dystrophy, suggesting that juvenile PM is extremely uncommon, if it exists at all [17].

In the case of dysferlinopathy, most patients have muscle biopsies with an increased inflammatory response [20, 21], even those who are clinically less affected, suggesting that this manifestation is a relatively early feature [22]. Therefore, dysferlinopathy patients are most vulnerable to misdiagnosis with PM. Indeed, in an international multicenter study that included 193 patients, 16% with dysferlinopathy were misdiagnosed with PM [23], and another 10 of 40 patients (25%) were likewise misdiagnosed in a study in two French neuromuscular centers [3]. A systematic review of the literature was performed in PubMed database to identify all relevant reports of dysferlinopathy misdiagnosed as polymyositis. The key search terms included “dysferlinopathy”, “polymyositis”, “inflammatory myopathy”, “case report” & “misdiagnosis”. All case reports and case series of patients with dysferlinopathy published between 1999 and 2021 were eligible for inclusion. There were no language restrictions in the searching. Based on these criteria, a total of 20 studies meet the selection criteria giving a total of 32 dysferlinopathy cases [16, 20, 22, 24–40], reported as case reports or small case series, that were misdiagnosed with PM (Table 1).

**Table 1:** Review of reported cases of dysferlinopathy misdiagnosed with polymyositis

Patient	Sex	Age at onset (years)	Age at diagnosis (years)	Time to diagnosis (years)	Initial diagnosis	Treatment	Outcome	Dysferlinopathy diagnosis			Phenotype	Reference
								IHC	WB	MD		
1	Male	23.5	25	1.5	IM	NS	NS	−	−	−	MMD1	[22]
2	Female	18	29	11	PM	CCS	No response	−	−	−	MMD1	[22]
3	Male	17	NS	NS	PM	CCS	No response	−	+	+	MMD1	[20]
4	Male	17	NS	NS	PM	CCS	No response	−	+	+	MMD1	[20]
5	Female	32	NS	NS	PM	CCS	No response	−	−	+	LGMDR2	[20]
6	Male	17	NS	NS	PM	CCS	No response	−	+	+	LGMDR2	[20]
7	Female	32	NS	NS	PM	CCS	No response	−	+	+	LGMDR2	[20]
8	Male	17	45	28	PM	NS	NS	−	−	+	MMD1	[24]
9	Female	26	30	4	IM	NS	NS	−	−	+	MMD1	[24]
10	Male	24	33	9	PM	CCS, AZA	No response	+	+	−	LGMDR2	[25]
11	Female	Third decade	55	NS	PM	CCS, AZA	No response	+	−	−	LGMDR2	[26]
12	Female	16	21	5	PM	CCS, IVIg, MTX, IFX	No response	+	+	+	LGMDR2	[27]
13	Female	27	38	11	PM	CCS, IVIg, MTX, CYC, CSA	Worsened	+	+	+	LGMDR2	[16]
14	Male	15	20	5	IM	CCS, AZA, IVIg	No response	+	+	+	MMD1	[28]
15	Male	14	17	3	PM	CCS, AZA, CSA, IVIg	N0 response	−	+	+	NS	[29]
16	Female	18	NS	NS	IM	CCS, IMMs	NS	+	−	−	LGMDR2	[30]
17	Female	33	NS	NS	IM	CCS, IMMs	NS	+	−	−	LGMDR2	[30]
18	Male	21	NS	NS	IM	CCS, IMMs	NS	+	−	−	MMD1	[30]
19	Male	20	NS	NS	IM	CCS, IMMs	NS	+	−	−	MMD1	[30]
20	NS	NS	NS	NS	PM	CCS	NS	+	+	+	MMD1	[31]
21	Female	50	54	4	PM	CCS	No response	+	−	−	LGMDR2	[32]
22	Female	35	40	5	PM	CCS, MTX, AZA, MMF, RTX	No response	−	+	+	LGMDR2	[33]
23	Male	37	NS	NS	PM	CCS, CYC, MTX, AZA, IVIg	Progressed	+	−	−	LGMDR2	[34]
24	Female	17	NS	NS	PM	CCS	No response	−	−	+	LGMDR2	[35]
25	Female	15	18	3	PM	CCS, MTX, IVIg	Minimal improvement	−	−	+	MMD1/ LGMDR2	[35]
26	Male	23	NS	NS	PM	CCS, IVMPD, MTX, AZA, IVIg	No response	−	+	+	MMD1/ LGMDR2	[35]
27	Female	32	NS	NS	PM	CCS, MTX, AZA	Worsened	−	−	+	MMD1/ LGMDR2	[35]
28	Male	37	37	0	IM	DEX	No response	+	-	-	MMD1	[36]
29	Male	16	19	3	PM	CCS	Progressed	−	−	+	MMD1	[37]
30	Female	25	42	17	PM	CCS, MTX, AZA, CSA, MMF, IVIg, RTX	Progressed	−	−	+	LGMDR2	[38]
31	Female	18	23	5	PM	CCS, IMMs	Worsened	+	+	+	LGMDR2	[39]
32	Female	22	3 months after onset	0.25	IM	CCS,MTX	Progressed	+	−	+	LGMDR2	[40]

*NS* not specified; *PM* polymyositis; *IM* inflammatory myopathy; *CCS* corticosteroids; *AZA* azathioprine; *IVIg* intravenous gamma globulin; *MTX* methotrexate; *IFX* infliximab; *CYC* cyclophosphamide; *CSA* cyclosporine; *IMMs* immunosuppressants; *RTX* rituximab; *IVMP* intravenous methylprednisolone; *MMF* mycophenolate mofetil; *IHC* immunohistochemistry; *WB* Western blot; *MD* molecular diagnosis; *MMD1* miyoshi muscular dystrophy-1; *LGMDR2* limb girdle muscular dystrophy 2B; *DEX* dexamethasone

Of these, 55% were female, the median age at onset of symptoms was 21.5 years (range, 14–50 years), and onset in 33% of cases was before age 18 years. The median time elapsed for the diagnosis was 5 years (range, 0–28 years), and the LGMDR2 phenotype was reported in 57% (16/28). Thus, dysferlinopathy can be diagnostically challenging because of its considerable genetic and phenotypic heterogeneity and clinical and histological characteristics that overlap with IIM.

Establishing an accurate differential diagnosis is imperative not only to guide treatment, prognosis, and genetic counseling but also to prevent unnecessary and potentially harmful treatment. One case series of 20 patients with dysferlinopathy who were initially misdiagnosed as having inflammatory myopathy showed that muscular strength may worsen after corticosteroid treatment and might not be regained after cessation of corticosteroids [14]. Likewise, in a randomized controlled trial with deflazacort, dysferlinopathy patients did not improve during the treatment period, and there was a trend to worsening in muscle strength [41]. Here, we report a case of a 14 years-old male patient with dysferlinopathy and pre-symptomatic hyperCKemia, in whom muscular weakness was triggered by corticosteroid treatment for misdiagnosed juvenile PM. Furthermore, he did not experience full muscular strength recovery after stopping treatment.

Although based on direct peer guidelines for asymptomatic hyperCKemia it was more likely to be dystrophinopathy [42], this was discarded by direct genetic testing. Next-generation sequencing techniques provide a potential way to overcome diagnostic delays. WES yields a higher diagnostic rate than sequential genetic testing for undiagnosed patients with limb-girdle weakness [43]. In our patient, two previously described *DYSF* mutations [3, 44], were detected by WES. Both variants cause a truncated version of the protein. Although immunodetection on muscle biopsies has shown that dysferlinopathy represent the second largest proportion of rare muscular dystrophies (18.45%) after dystrophinopathies (52.3%) in Mexico [45], the characterization of *DYSF* mutations is scarce [46]. In fact, the c.3851C > T mutation has been described only in one Mexican MMD1 patient, and although the c.5979dup mutation has been characterized as frequent [47], this is the first report in a Mexican patient. Furthermore, neither of these mutations was found in 2217 exomes from Mexican volunteers from a previous study [48], although we found seven variants predicted to be pathogenic and described in the Mexican population for the first time (Table 2). Thus, personalized and precision medicine is critical in highly heterogeneous diseases such as IIM and LGMD. In line with this, some therapeutic approaches should be considered, such as the use of antisense-induced exon skipping, which has shown promising results for *DYSF* exon 32 skipping. The deletion of this exon produces a mild phenotype, making this exon suitable for exon skipping [49].

**Table 2:** Most frequent variants with clinical implications identified from 2217 exomes from Mexican Amerindian and Mestizo individuals

Variant	Genomic position	Allele change	MAF	Gene location	Mutation type	Nucleotide/Aminoacid Change	Pathogenecity (ClinVar/InterVar)
–	71,896,854	delA	0.00045	Exonic	Frameshift deletion	c.5645delA (p.V1883Sfs*83)	clinvar: Pathogenic/InterVar: Pathogenic
rs115407852	71,908,183	G/A	0.00045	Exonic/Ferlin, C-terminal domain	Nonsynonymous SNV	c.6116G > A (p.Arg2039Gln)	clinvar: Conflicting_interpretations_of_pathogenicity/InterVar: Likelypathogenic
–	71,783,202	G/A	0.00023	Intronic	Splicing	–	clinvar: UNK/InterVar: Pathogenic
rs863225021	71,892,311	C/T	0.00023	Exonic	Nonsynonymous SNV	c.5194C > T (p.Arg1732Trp)	clinvar: Pathogenic/Likely_pathogenic/InterVar: Uncertainsignificance
rs746243052	71,894,607	C/T	0.00023	Exonic/C2 domain	Nonsynonymous SNV	c.5419C > T (p.Arg1807Trp)	clinvar: Pathogenic/Likely_pathogenic/InterVar: Uncertainsignificance
rs121908955	71,909,727	C/T	0.00023	Exonic/Ferlin, C-terminal domain	Nonsynonymous SNV	c.6241C > T (p.Arg2081Cys)	clinvar: Pathogenic/Likely_pathogenic/InterVar: Likelypathogenic
rs34061568	71,797,381	A/C	0.00023	Exonic/Peroxin/Ferlin domain	Nonsynonymous SNV	c.3002A > C (p.Lys1001Thr)	clinvar: Conflicting_interpretations_of_pathogenicity/InterVar: Likelypathogenic

In conclusion, distinguishing an inflammatory myopathy from dysferlinopathy is quite difficult if diagnosis is based only on routine clinicopathological examination. We suggest that before establishing a diagnosis of “definite” or “probable” juvenile PM, according to Bohan and Peter or ACR/EULAR criteria, muscular dystrophy with inflammatory characteristics on muscle biopsy must first be ruled out. For an accurate diagnosis, immunohistochemistry or Western blot analysis should be applied to identify reduction or loss of protein, and/or genetic analysis by WES applied to identify mutations and rule out other muscular dystrophies. These steps, along with a strategy for approaching the history and examination of patients with hyperCKemia may help the clinician to identify the etiology of hyperCKemia and prevent more misdiagnoses and inappropriate treatment with steroids and immunosuppressors in patients with dysferlinopathy.

## Data Availability

The variant data for this study have been deposited in the European Variation Archive (EVA) at EMBL-EBI under accession number PRJEB53236. (https://www.ebi.ac.uk/eva/?eva-study=PRJEB53236). The data concerning the *DYSF* variants in 2217 exomes from Mexican volunteers were obtained from a previous study [48].

## Abbreviations

LGMD: Limb-girdle muscular dystrophy
CK: Creatine kinase
MMD1: Miyoshi muscular dystrophy-1
PM: Polymyositis
EMG: Electromyography
IVMP: Intravenous methylprednisolone
WES: Whole-exome sequencing
IIMs: Idiopathic inflammatory myopathies
